# Cellular Mechanisms of Cortisol-Induced Changes in Mauthner-Cell Excitability in the Startle Circuit of Goldfish

**DOI:** 10.3389/fncir.2017.00068

**Published:** 2017-09-28

**Authors:** Daniel R. Bronson, Thomas Preuss

**Affiliations:** ^1^The Graduate Center, City University of New York, New York, NY, United States; ^2^Hunter College, City University of New York, New York, NY, United States

**Keywords:** cortisol, Mauthner-cell, *in vivo* electrophysiology, hormones, input resistance, voltage-gated ion channels, excitability, startle

## Abstract

Predator pressure and olfactory cues (alarm substance) have been shown to modulate Mauthner cell (M-cell) initiated startle escape responses (C-starts) in teleost fish. The regulation of such adaptive responses to potential threats is thought to involve the release of steroid hormones such as cortisol. However, the mechanism by which cortisol may regulate M-cell excitability is not known. Here, we used intrasomatic, *in vivo* recordings to elucidate the acute effects of cortisol on M-cell membrane properties and sound evoked post-synaptic potentials (PSPs). Cortisol tonically decreased threshold current in the M-cell within 10 min before trending towards baseline excitability over an hour later, which may indicate the involvement of non-genomic mechanisms. Consistently, current ramp injection experiments showed that cortisol increased M-cell input resistance in the depolarizing membrane, i.e., by a voltage-dependent postsynaptic mechanism. Cortisol also increases the magnitude of sound-evoked M-cell PSPs by reducing the efficacy of local feedforward inhibition (FFI). Interestingly, another pre-synaptic inhibitory network mediating prepulse inhibition (PPI) remained unaffected. Together, our results suggest that cortisol rapidly increases M-cell excitability via a post-synaptic effector mechanism, likely a chloride conductance, which, in combination with its dampening effect on FFI, will modulate information processing to reach threshold. Given the central role of the M-cell in initiating startle, these results are consistent with a role of cortisol in mediating the expression of a vital behavior.

## Introduction

Acute stress broadly promotes behaviors that increase the likelihood of survival in part through the release of steroid hormones that include corticosteroids (McEwen, 1998; Sapolsky, 2002). There is growing evidence that corticosteroids influence the excitability of neurons and mediate rapid changes in behavior (Orchinik et al., 1991; Rose et al., 1998; Remage-Healey and Bass, 2004). For example, *in vitro* studies in rodents support that corticosteroids interact with neurotransmitters such as glutamate, serotonin and GABA to modulate sensory integration in the amygdala and hippocampus such that awareness to the environment is increased via a change in sensory gain (Stutzmann et al., 1998; Venero and Borrell, 1999; Karst et al., 2005, 2010; Groc et al., 2008). However, how these changes in sensory gain translate directly to behavioral output measures remains an important question. The vertebrate acoustic startle network is well-suited to study modulatory effects of corticosteroids on behavior since it is mediated by relatively few neurons that produce a distinct and quantifiable behavior (Eaton et al., 1977; Lingenhöhl and Friauf, 1992; Korn and Faber, 1996; Koch, 1999; Weiss et al., 2006; Fetcho, 2009). In addition, startle has been shown to be sensitive to acute administration of corticosteroids in many vertebrates (Fehm-Wolfsdorf et al., 1993; Sandi et al., 1996; Buchanan et al., 2001; Richter et al., 2011).

The startle escape response of teleost fish, or C-start, can be modified by environmental threats that putatively involve the release of steroid hormones. Cortisol has been shown to peak within minutes of exposure to stressors (Wendelaar Bonga, 1997; Flik et al., 2006). Stressors that are thought to increase C-start responsiveness include increased perceived threat from predators (Domenici, 2010; Fischer et al., 2014, 2015) and alarm substance (Speedie and Gerlai, 2008). The goldfish startle circuit consists of two large reticulospinal neurons, the Mauthner cells (M-cells) that integrate multimodal sensory input and a single action potential (AP) in either M-cell triggers the C-start by activating contralateral spinal musculature (Zottoli, 1977; Eaton et al., 1981; Faber et al., 1989; Nissanov et al., 1990; Fetcho, 1991, 2009). Therefore, M-cell excitability is directly correlated with changes in the startle responsiveness, and are a predictor of behavioral output (Preuss and Faber, 2003; Korn and Faber, 2005; Neumeister et al., 2008; Curtin et al., 2013). Accordingly, M-cells have been employed to study startle plasticity and sensorimotor gating in goldfish (Medan and Preuss, 2011; Curtin et al., 2013) African cichlids (Neumeister et al., 2010, 2017; Whitaker et al., 2011) and zebrafish (Burgess and Granato, 2007).

M-cell activation depends on massive excitation that is opposed by tonic and feed-forward networks, which include modulation through dopaminergic and serotonergic inputs (Furshpan and Furukawa, 1962; Faber and Korn, 1982; Pereda et al., 1992; Medan and Preuss, 2011; Curtin et al., 2013). As such, M-cell excitability is sensitive to changes in physiological state and environmental stressors such as predator pressure and social defeat (Neumeister et al., 2010, 2017; Whitaker et al., 2011; Fischer et al., 2014). Thus, here we asked if cortisol could mediate such changes at the level of the M-cell. Our results indicate that cortisol increases M-cell excitability thus modulating the time-course of auditory-evoked post-synaptic potentials (PSPs).

## Materials and Methods

### Subjects

Adult goldfish (*Carassius auratus*; *N* = 17) of either sex were purchased from Ozark Fisheries (Stoutland, MO, USA). Fish measured 7–10 cm in body length and were housed in groups of 5–10 in rectangular plexiglass holding tanks (30 × 30 × 60 cm; 95 L). Tanks were supplied with recirculating and filtered conditioned water maintained at 18°C. Water was conditioned as described previously (Szabo et al., 2006). Ambient light was set to a 12 h light/dark photoperiod.

### Pharmacology

In Experiment 1, the control condition consisted of a bath solution consisting of saline (modified Cortland’s solution, in mM: 124.0 NaCl, 5.1 KCL, 2.8 monobasic NaH_2_PO_4_H_2_O, 0.9 anhydrous MgSO_4_, 20.0 HEPES, 1.6 CaCl_2_·2H_2_O, 5.6 dextrose buffered to 7.2 pH) and 0.1% Tween-20 that was superfused directly onto the exposed medulla via a stainless steel ball-tipped perfusion needle that was stabilized above the brain and attached to a tube and syringe needle. After baseline measurements in the control condition, the bath solution was replaced with cortisol (hydrocortisone, Sigma Aldrich) dissolved in 500 μL solution consisting of saline and 0.1% Tween-20 at a final cortisol concentration of 8 mM (*N* = 10). Superfusion is a well-established method to assess drug effects on M-cell activity (Pereda et al., 1992; Medan and Preuss, 2011; Curtin and Preuss, 2015), which has been successfully linked to changes in startle behavior (Curtin et al., 2013). Previous studies that superfuse of drugs onto the medulla estimate that the diffusion of the drug through the brain reduces the final concentration at the level of the M-cell (1.5 mm below the surface of the brain) by at least 2 orders of magnitude (Pereda et al., 1992, 1994). Accordingly, the concentration of cortisol was selected to achieve a concentration similar to *in vitro* studies that demonstrate acute effects of cortisol on neural activity (10^−6^ M in Hua and Chen, 1989, 10^−6^ M in Zaki and Barrett-Jolley, 2002). In addition, a series of control experiments compared M-cell activity with saline control and 0.1% Tween-20 dissolved in saline applied directly to the brain and found no effect on M-cell activity (*N* = 3, data not shown).

In Experiment 2, cortisol was injected IM in the caudal part of the fish body (600 mg/kg body weight, *n* = 7) at volumes that never exceeded 200 μL. This dose was selected to maximize saturation of the effect of cortisol for the longest time possible to accommodate our long-lasting (1 h) recording and stimulation protocol necessary to assess membrane properties. This dose was sufficient to observed the onset of effects emerge within 10 min and begin to return to baseline within an hour, which suggests that the M-cell activity remained physiologically stable following this high dose (see “Results” Section). We found no effect of route of drug administration on recording condition (Change in resting membrane potential (RMP), direct = −0.5 mV ± 1.4, systemic = 1.3 mV ± 1.2 *p* > 0.05), although data from Experiments 1 and 2 are discussed separately in the “Results” Section.

### Electrophysiology

*In vivo* surgery and electrophysiological recording was performed as previously described (Medan and Preuss, 2011; Curtin et al., 2013). Briefly, subjects were immersed in ice water for 10–15 min before surgical procedures. Fish were then placed in the recording chamber, stabilized with one steel pin on each side of the head, and ventilated through the mouth with recirculating, aerated conditioned water at 18°C. In Experiment 1, the general anesthetic MS-222 was dissolved in the recirculating water at a dosage (20 mg/L, *N* = 10) that does not interfere with auditory processing (Palmer and Mensinger, 2004; Cordova and Braun, 2007). In Experiment 2, the opioid agonist fentanyl (1 mg/kg, *N* = 7) was injected IM as an alternative analgesic at dosages similar to or less than those that have demonstrated no effect on auditory processing (Cordova and Braun, 2007; Neiffer and Stamper, 2009). The recording chamber was mounted inside an opaque, thin-walled tank filled with temperature controlled (18°C) saline covering the fish body up to the midline. Next, the spinal cord was exposed with a small lateral incision at the caudal midbody. Bipolar electrodes were placed on the spinal cord to transmit low-intensity (5–8 V) electrical stimulation generated by an isolated stimulator (Digitimer). Antidromic activation of the M-cell axons was confirmed by a visible muscular contraction (twitch). Goldfish were then injected intramuscularly with either D-tubocurarine (1 μg/g body weight; Abbott Laboratories) or flaxedil (1 μg/g body weight; Sigma Aldrich). A small craniotomy was performed and the medulla was exposed for intracellular. Antidromic stimulation produces a negative potential in the M-cell axon cap (typically 15–20 mV) that unambiguously identifies the axon hillock and allows intracellular recordings from defined locations along the M-cell soma–dendritic membrane (Furukawa and Ishii, 1967; Faber et al., 1989). The sharp electrodes were therefore guided electrically to the all-or-none field potentials that are evoked by M-cell spikes, which occur with a latency of approximately 30–40 μs in response to anti-dromic stimulation. Intracellular recording of M-cell responses to sound stimuli were acquired using an Axoprobe-1A amplifier (Molecular Devices) in current-clamp mode with sharp electrodes (5–9 MΩ) filled with 5 M potassium acetate (KAc). Recordings were stored online with a Macintosh G5 computer using a data acquisition card (PCI-E; National Instruments) sampling at 25 kHz. Sound stimuli consisted of single-cycle sound pips (200 Hz) produced by a function generator (Agilent 33210A) connected to a shielded subwoofer (Behritone C50A; Behringer) located 30 cm from the recording chamber. Reported dB values are presented as Sound Pressure Levels (SPL) with a hydrophone at a reference value of 1 μPa instead of the typical references value in air of 20 μPa, which corresponds to the human hearing threshold. Thus, the dB values in water correspond to an approximately 62 dB increase relative to recordings in air. Sound stimuli were recorded with a microphone placed within 10 cm of the fish’s head. Values from five individual traces from each stimulus condition were averaged and used for analysis.

The effect of cortisol on M-cell membrane properties including threshold current and input resistance (Neumeister et al., 2008; Medan and Preuss, 2011) was studied by injecting current ramps via a second intrasomatic electrode (KAc; 3–5 MΩ) while maintaining the voltage recordings. A function generator (model 39; Wavetek) was used to regulate current injection, producing a positive current ramp (0–240 nA/20 ms). A compensation circuit built in the Axoprobe-1A amplifier eliminated crosstalk between the electrodes. Current–voltage (I/V) relationships were measured without sensory stimulation or with an auditory prepulse (200 Hz, 139, 153, or 173 dB relative to 1 μPa in water) preceding current injection by 20, 50, 150, 200 or 500 ms. After assessment of baseline conditions, cortisol was administered either directly onto the brainstem via superfusion with Ringer’s saline or via IM injection (see above). Post-drug measures were taken after 10 mins and repeated for up to 2 h after injection. RMP was continuously monitored to ensure stable recording conditions and/or possible effects of cortisol. A typical experiment lasted 4–5 h.

This study was carried out in accordance with the recommendations of the Hunter College (City University of New York) Institutional Animal Care and Use Committee. The protocol was approved by the Hunter College (City University of New York) Institutional Animal Care and Use Committee. Data were analyzed with JMP 12.0 (SAS Institute), and figures were created in Igor Pro (version 5.03; Wavemetrics). Data presented in figures describe mean values, and error bars illustrate mean standard error (SEM). The Shapiro-Wilk test was used to confirm that datasets met assumptions of normality.

Hypothesis testing was performed using matched *t*-tests before and after cortisol, with a significance threshold of 0.05, *α* = 0.05. Effect sizes were measured using Cohen’s d and corrected for dependence of means for within comparisons per Morris and DeShon (2002). The effect of cortisol on prepulse inhibition (PPI) in Experiment 2 only was analyzed using a two-way repeated measures analysis of variance (ANOVA) with subjects as repeated measures, interstimulus interval (ISI20 ms, ISI50 ms, ISI150 ms, ISI200 ms, ISI500 ms) or sound intensity (139, 153, 173 dB relative to 1 μPa in water), and drug (saline, CORT) condition as factors, and the change in threshold current was the dependent variable. These analyses were followed up Bonferroni corrected *post hoc* tests, when indicated. The effect size for each factor was quantified using the partial eta squared.

## Results

### Experiment 1—Effects of Cortisol on Auditory-Evoked M-Cell Responses

**Figure 1:**
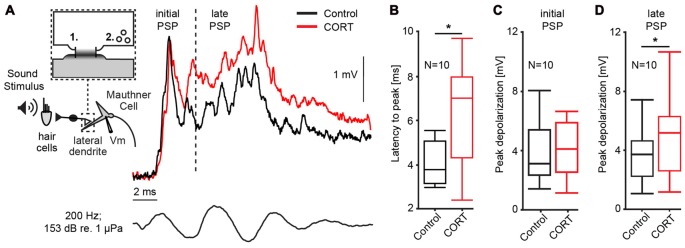
Cortisol potentiates M-cell synaptic sound response. **(A)** *Inset*: schematic of 8th nerve inputs to the M-cell. Eighth nerve afferences project onto lateral dendrite via large myelinated club endings that contain: (1) gap junctions; and (2) glutamatergic synapses. Membrane voltage (Vm) was recorded in the soma or proximal dendrite during sound stimulation. To the right, example trace of sound-evoked post-synaptic potentials (PSPs) recorded in the M-cell before (black) and after (red) treatment with cortisol. Bottom trace indicates sound stimuli. Dashed line indicates 5 ms latency after stimulus onset; to the left, the sound response consists of excitatory, electrotonic components only (initial PSP), whereas to the right of the line (late PSP) the response also includes mixed excitatory and inhibitory chemical components. **(B)** Boxplots of mean latency-to-peak (ms) of sound-evoked PSPs (*N* = 10) for control and CORT treatment. Paired *t*-test, *p = 0.0195. **(C)** Boxplots of mean peak amplitudes of the initial PSPs (*N* = 10) for control and CORT treatment. Paired *t*-test, *p* = 0.112. **(D)** Boxplots of mean peak amplitudes of the initial PSPs (*N* = 10) for control and CORT treatment. Paired *t*-test, *p = 0.012.

Our first set of experiments (*N* = 10) characterized the effect of cortisol on the auditory response in the M-cell, the decision-making neuron in the goldfish startle circuit. The M-cell receives di-synaptic inputs from inner ear hair cells via numerous 8th nerve afferences that converge onto the distal lateral dendrite via mixed electric (gap junctions) and chemical synapses (Bodian, 1952; Lin and Faber, 1988a; Pereda et al., 2003). Thus, responses to sound pips reflect initially (<5 ms after sound onset) electrotonic coupling potentials only, whereas later parts of the response also involve excitation by chemical synapses (Szabo et al., 2006). Moreover, excitation is counteracted by feedforward inhibition (FFI) approximately 5 ms after sound onset (Preuss and Faber, 2003; Korn and Faber, 2005; Weiss et al., 2009). As such, the M-cell PSP can be dissected into an initial excitatory PSP that essentially reflects AP events in the presynaptic afferences (defined as initial PSP in Figure 1A), whereas the later PSP represents an interaction of excitatory and inhibitory inputs via chemical synapses (defined as late PSP in Figure 1A; Szabo et al., 2006).

PSPs were recorded in the M-cell soma in response to a sound pip (153 dB relative to 1 μP in water) before and after superfusing cortisol (8 mM) onto the surface of the medulla. In Figure 1A, representative individual traces before (black) and after drug (red) show that cortisol changed the waveform of the PSP namely, it enhanced the later part of the response indicted by a shift in latency-to-peak (i.e., the time from sound onset to peak response) and an increase in peak amplitude. To quantify these effects, we compared the latency-to-peak (ms), as well as the initial and late PSP peak amplitudes (mV) from averaged traces (5) before and after cortisol treatment (Figures 1B–D). Latency-to-peak increased by 51.58% ± 22.68 after cortisol, which is a large and significant effect (mean control: 4.25 ± 0.31 ms; mean drug: 6.21 ± 0.71 ms; paired *t*-test, *t* = 8.05, *p* = 0.0195; *d* = 1.00, *N* = 10; Figure 1B). Amplitude measurements showed a large increase in initial PSP peak size (12.92 ± 7.05%), but this difference was not statistically significant (mean control: 3.74 ± 0.61 mV; mean drug: 4.52 ± 1.02 mV; paired *t*-test, *t* = 3.12, *p* = 0.112; *d* = 1.38, *N* = 10, Figure 1C). However, cortisol had a large effect on peak amplitude, which was significantly increased for the late PSP (28.26 ± 7.46% increase, mean control: 3.62 ± 1.80 mV; mean drug: 4.69 ± 2.51 mV; paired *t*-test, *t* = 9.73, *p* = 0.0123, *d* = 1.31, *N* = 10, Figure 1D). Cortisol did not change the M-cell RMP (RMP_control_ = −82.16 ± 0.87 mV; RMP_drug_ = −82.68 ± 1.38 mV; paired *t*-test, *t* = 0.23, *p* = 0.64, *d* = 0.287, *N* = 10).

**Figure 2:**
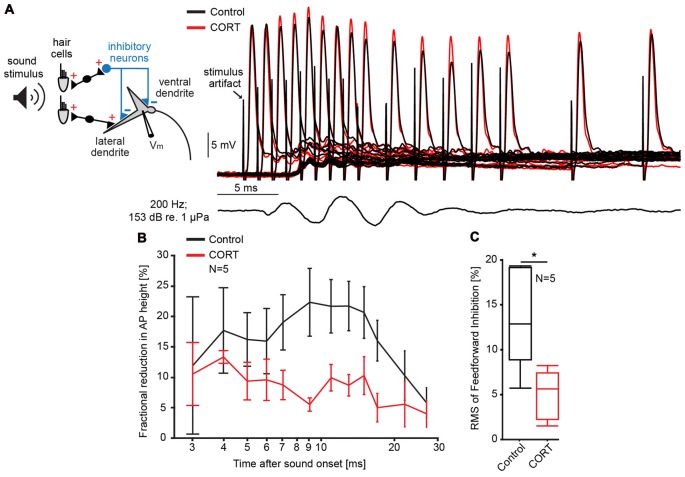
Cortisol attenuates feedforward inhibition (FFI) in the M-cell. **(A)** *Inset*: schematic of M-cell and associated excitatory and feedforward inhibitory networks. FFI is quantified as the fractional amplitude reduction of antidromically evoked test action potentials (APs) concurrent with sound stimulation. To the right, recordings of APs in control (black) and CORT (red) conditions for sound/AP intervals ranging from 1 ms to 30 ms. Bottom trace indicates sound stimuli (200 Hz, pips at 153 dB relative to 1 μPa in water). **(B)** Time-course of FFI expressed as the mean fractional reduction (± SEM, *N* = 5) of the test AP before (black) and after (red) CORT. **(C)** Boxplots of the mean root mean square (RMS) values of the evoked FFI in control (black) and drug trials (paired *t*-test, *p = 0.0391; *N* = 5).

One possible mechanism underlying the cortisol-induced potentiation of the late PSP is a reduction in FFI. Inhibition in the M-cell is of the shunting type and related to changes in the cell’s input resistance (Korn and Faber, 1976, 2005). Thus, the magnitude and duration of sound evoked inhibition (inhibitory time-course) can be quantified in the M-cell as the fractional amplitude reduction of an antidromically test AP in response to a conditioning sound stimulus that precedes the AP at distinct time intervals (Figure 2A; Faber and Korn, 1982). Figure 2B shows the mean inhibitory shunt (defined as 100 − AP_test_/AP_control_ *100; Faber and Korn, 1982) at sound onset/AP intervals ranging from 2 ms to 50 ms in five M-cells. For quantification, we calculated the root mean square (RMS) values of the fractional shunt of the AP over the entire range. The results suggest that cortisol reduced the overall magnitude of FFI (paired *t*-test, *t* = 9.12, *p* = 0.0391; *d* = −1.41, *N* = 5, Figure 2C) while the inhibitory time-course was still intact (Figure 2B). Together, the results suggest that reduced FFI might be at least partly responsible for the potentiation of the late PSP after cortisol application (Figure 1A).

### Experiment 2—Effects of Cortisol on M-Cell Membrane Properties and PPI

**Figure 3:**
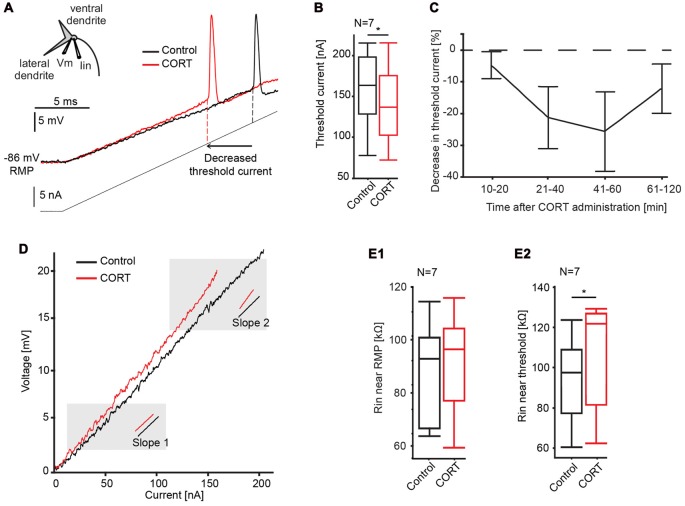
Effects of cortisol on M-cell membrane properties. **(A)** *Inset*: schematic of M-cell. A current-injecting electrode (Iin) is placed in the soma while voltage (Vm) is recorded in the proximal dendrite. Traces show the M-cell membrane response to a current ramp injection (240 nA, 20 ms, gray line) before (black trace) and after (red trace) CORT. Dashed lines indicate threshold current in control and drug conditions. **(B)** Boxplots of mean threshold currents in control (black) and drug (red) conditions (paired *t*-test, *p = 0.0307, *N* = 7). **(C)** Time-course of CORT effects. Plot of normalized changes in threshold current at distinct time windows following drug injection (Means ± SE, *N* = 6, 6, 5 and 4 for the 10–20, 21–40, 41–60, 61–120 min windows, respectively). Dotted line represents no change in threshold current. **(D)** Sample voltage/current plot before (black) and after (red) drug. M-cell input resistance near resting membrane potential (RMP) was largely unaffected by CORT (compare slopes at 2 mV above RMP). Resistance near threshold was increased near threshold (compare slopes at 2 mV below threshold). **(E)** Boxplots of mean M-cell input resistance (R_in_) near RMP **(E1)** and in the depolarized M-cell membrane **(E2)** (paired *t*-test, *p* = 0.0187, *N* = 7).

To test effects of cortisol on the postsynaptic M-cell membrane directly, we injected a 20 ms current ramp into the M-cell soma (*M* = 160 ± 16.2 nA) while recording membrane voltage with a second electrode (Figure 3A). This technique allows us to measure changes in membrane properties such as threshold current, absolute threshold and voltage-dependent conductances over a wide-range of membrane depolarizations, including inhibitory conductances evoked during PPI (Neumeister et al., 2008; Medan and Preuss, 2011; Curtin et al., 2013). Here, cortisol was administered systemically as PPI neurons are extrinsic to the M-cell startle circuit in the hindbrain (Bergeron et al., 2015). We found that cortisol reduced the amount of current necessary to fire the cell in six of the seven recorded M-cells. The quantification of this effect (see “Materials and Methods” Section) showed a reduction in threshold current by 9.17 ± 3.05% (control: 157.12 ± 18.20 nA, drug: 143.04 ± 18.15 nA; paired *t*-test, *t* = 7.91, *p* = 0.0307, *d* = 1.063, *N* = 7, Figure 3B). In contrast, absolute threshold (control: −70.43 ± 1.95 mV; drug: −70.0 ± 2.46 mV, paired *t*-test, *t* = 0.1358, *p* = 0.7252, *d* = 0.154, *N* = 7), and RMP (control: −85.41 ± 1.54 mV; drug: −84.10 ± 2.11 mV, paired *t*-test, *t* = 1.0119, *p* = 0.3533, *d* = 0.416, *N* = 7) were unchanged. Since these experiments did not involve activation of any presynaptic networks, the results are consistent with an excitatory action of cortisol at the postsynaptic M-cell membrane.

To assess the pharmacokinetics of systemically-administered cortisol, its effect on threshold current was quantified at four distinct post-injection time windows between 10–20, 21 and 40, 41 and 60, and 61 and 120 min. The results indicated a distinct drug effect between 10 min and 60 min, before returning to baseline (Figure 3C). Therefore, threshold current measurements within the 10–60-min period were utilized for data analysis.

The current-voltage relationship was further analyzed for effects of cortisol on M-cell input resistance. The M-cell is a high-threshold neuron with relatively low-input resistance at baseline, however resistance increases dynamically when membrane depolarization exceeds approximately five mV above RMP (Faber and Korn, 1986; Neumeister et al., 2008). This membrane nonlinearity is caused by a membrane conductance (putatively an inward rectifier potassium channel) that inactivates in the depolarizing cell, which functionally increases M-cell excitability, i.e., it acts as a sensory high pass filter (Faber and Korn, 1986). Thus, to dissect the action of cortisol on the post-synaptic membrane, we measured M-cell input resistance derived as the slope in I/V plots close to RMP (slope 1, Figure 3D) and in the depolarized cell (slope 2, Figure 3D; see “Materials and Methods” Section for details). Cortisol did not change input resistance near RMP (slope 1) compared to control condition (control, mean = 88.2 ± 7.0 kΩ, drug, mean = 93.4 ± 8.3 kΩ; paired *t*-test, *t* = 1.2828, *p* = 0.3006, *d =* 0.433, *N* = 7, Figure 3E1), however, cortisol increased input resistance near threshold (slope 2; control, mean = 93.4 ± 8.3 kΩ, drug, mean = 105.3 ± 10.0 kΩ; paired *t*-test, *t* = 10.2143, *p* = 0.0187, *d =* 1.350, *N* = 7, Figure 3E2). These results indicate that the increase in M-cell excitability by cortisol is at least partly driven by the inactivation of a voltage-sensitive conductance.

Given the effects of cortisol on the M-cell membrane properties, as well as on FFI, we next asked if PPI, which is mediated by an extrinsic inhibitory network (Bergeron et al., 2015), was similarly affected. Interestingly, M-cell PPI is partly mediated by the activation of a voltage-sensitive membrane conductance (Neumeister et al., 2008; Medan and Preuss, 2011; Curtin et al., 2013). Thus, we tested whether the effects of cortisol on M-cell membrane nonlinearities potentiation described above interact or interfere with PPI.

**Figure 4:**
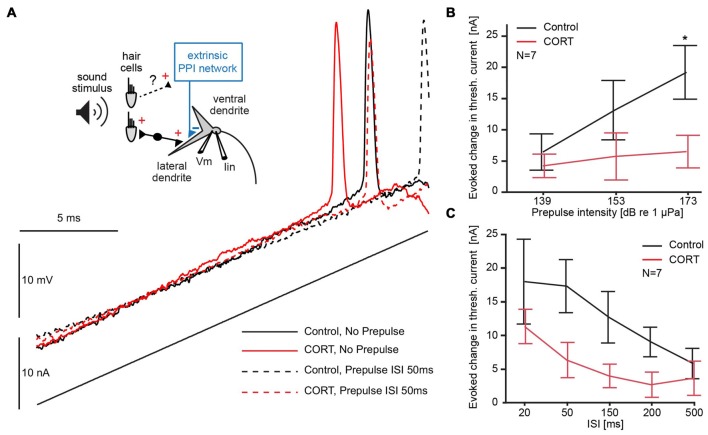
Testing the effects of cortisol on prepulse inhibition (PPI). **(A)** *Inset*: schematic of M-cell. 8th nerve afferences project to the M-cell and activate an extrinsic PPI network via an unknown pathway. A current-injecting electrode (Iin) is placed in the soma while voltage (Vm) is recorded in the proximal dendrite. Below and to the right, M-cell membrane response to a current ramp injection before (black) and after (red) CORT with (dashed lines) and without (solid lines) a 173 dB prepulse at 50 ms interstimulus interval (ISI). **(B)** Plots of the mean (± SEM, *N* = 7) evoked change in threshold current by a prepulse of three different intensities (50 ms prepulse/ramp ISI) in control (black) and CORT (red) conditions (Bonferroni *Post hoc*, *p = 0.0017). **(C)** Plots of the mean (± SEM, *N* = 7) evoked change in threshold current by a prepulse (173 dB) at indicated prepulse/ramp ISIs in control (black) and CORT (red) conditions. PPI was significantly greater at shorter ISIs (two-way repeated measures ANOVA, ISI, *F*_(4,61)_ = 5.23, *p* = 0.0011, *N* = 7). CORT reduced PPI across all ISIs (CORT, *F*_(1,61)_ = 35.71, *p* < 0.001, *N* = 7).

For this, we compared the membrane depolarization induced by current-ramp injections before (black) and after cortisol (red) with (dotted lines) and without (solid lines) a 173 dB prepulse at 50 ms ISI (Figure 4A). The results show an increase in threshold current in both drug and control conditions during PPI (Figure 4A; solid vs. dashed lines in Figure 4A), and an overall downshift in threshold current with and without prepulse following drug (Figure 4A; black vs. red traces).

To explore possible floor and ceiling effects, we assessed the effect of prepulse intensity on M-cell threshold current in drug and control trials at ISI 50, which has been shown to be an effective prepulse lead time in goldfish. Therefore, we used sound pips (200 Hz) of different intensities (139, 153, 173 dB relative to 1 μPa in water). Figure 4B shows the average increase in threshold current caused by prepulses of three different intensities at a lead time of 50 ms presented before the current ramp, demonstrating a positive relationship between prepulse intensity and threshold current (two-way repeated measures ANOVA, cortisol, *F*_(1,37)_ = 23.33, *p* < 0.001, partial eta squared = 0.387; prepulse intensity, *F*_(2,37)_ = 3.51, *p* = 0.0401, partial eta squared = 0.160, *N* = 7, Figure 4B). The effect of high-intensity prepulses on the increase in threshold current was significantly reduced after cortisol administration (Bonferroni *Post hoc*, *p* < 0.016).

To assess the effect of cortisol on the inhibitory time-course during PPI, high intensity (173 dB relative to 1 μPa) prepulse at five prepulse/current ramp ISIs of 20, 50, 150, 200 and 500 ms were applied. The results show that a prepulse evoked an increase in threshold current across all ISIs, but an overall downward shift of this effect in drug conditions (two-way repeated measures ANOVA, cortisol, *F*_(1,61)_ = 35.71, *p* < 0.001, partial eta squared = 0.370, *N* = 7, Figure 4C). The inhibitory PPI time-course, however, was still present after cortisol, indicated by an ISI-dependency of the evoked threshold effect in both drug and control conditions (ISI, *F*_(4,61)_ = 5.23, *p* = 0.0011, partial eta squared = 0.261, *N* = 7, Figure 4C). We found no significant interaction between cortisol and prepulse lead-time, suggesting no ISI-specific drug effects. Taken together, these results are indicative that cortisol leaves the PPI time-course largely intact but the effect of the prepulse evoked-inhibition is masked by an underlying tonic increase in excitability.

**Figure 5:**
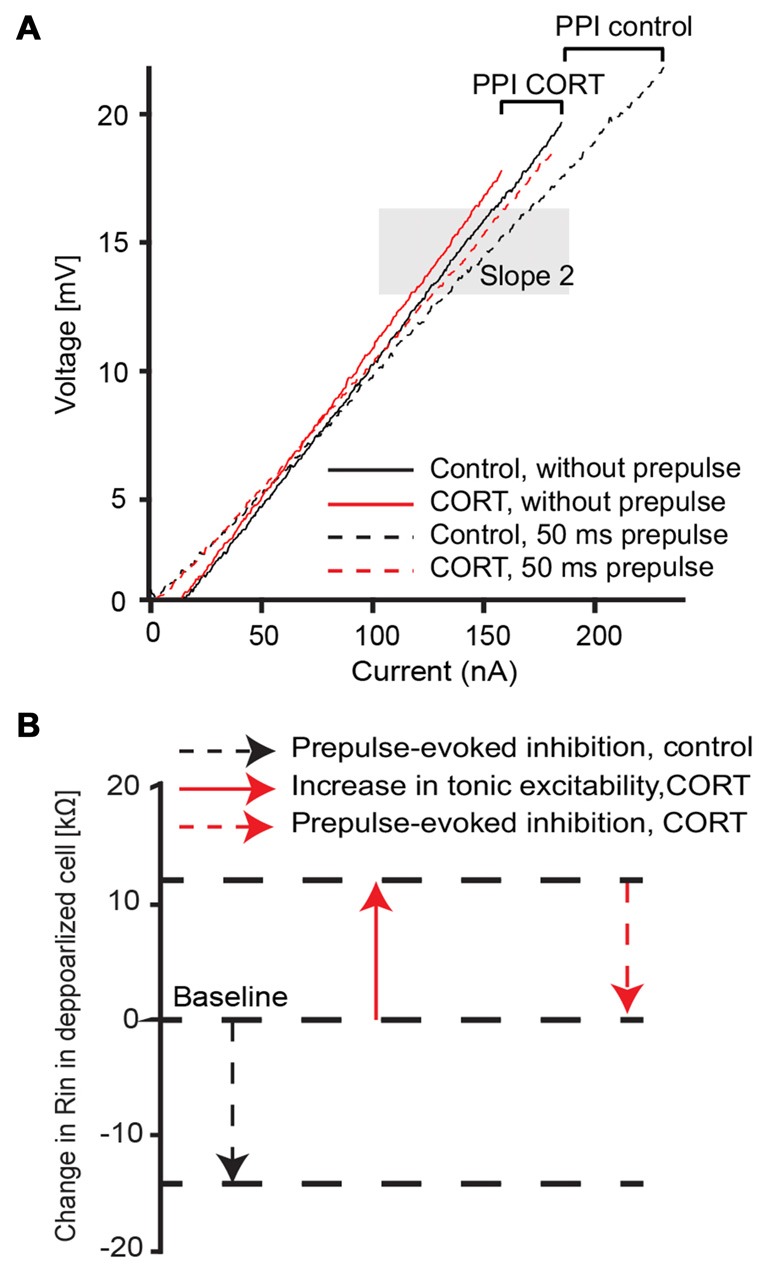
Cortisol effect on voltage-dependent membrane properties during PPI. **(A)** Average (five traces) voltage/current plot before (black) and after (red) drug and with (dashed lines) and without (solid lines) a 173 dB prepulse at 50 ms ISI, respectively. Shaded area depicts slope 2 assessment area in the depolarized membrane near threshold (slope 2). Brackets represent the evoked change in input resistance in control and drug condition. **(B)** Summary schematic of CORT induced effects on the depolarized M-cell membrane (slope 2 area in A) in control (black) and drug (red) conditions. Note: arrow length indicates mean (*N* = 7) values.

We next characterized the effect of cortisol on the voltage-sensitive conductance that mediates PPI by comparing the prepulse-evoked change of input resistance in the depolarized cell (slope 2, Figure 5A). Sample I/V plots before (black) or after (red) application of cortisol showed that a prepulse-evoked reduction in input resistance (high intensity prepulse, ISI 50 ms) in both control and drug conditions (Figure 5A, compare solid and dashed lines in shaded area). The results (*N* = 7) showed no significant difference in the prepulse-evoked effect on input resistance between control (mean prepulse-evoked decrease = 15.88 kΩ ± 2.9) and drug (mean prepulse-evoked decrease = 12.76 kΩ ± 2.6, paired *t*-test, *t* = 1.90, *p* = 0.21). However, the cortisol-evoked tonic increase in M-cell input resistance described above produces an overall shift in baseline excitability (Figure 5A, compare dashed black lines and solid red lines); thus, effectively generating the phenomenon such that excitability in PPI drug conditions is comparable to the no prepulse condition in controls. Figure 5B outlines the relative contribution (i.e., the means of seven experiments indicated by the length of the arrows) of cortisol induced-tonic increases in excitability that counteracts the evoked decrease in input resistance during PPI.

## Discussion

This study examined the role of the steroid hormone cortisol in regulating startle circuit excitability and sensory processing in the goldfish. *In vivo* intracellular recording found that cortisol acutely increases excitability of the startle decision-making neuron, the M-cell. Specifically, cortisol decreased auditory evoked FFI and caused a tonic voltage-dependent increase in M-cell input resistance but had no effect on PPI. Together, these effects contribute to a potentiation of the synaptic responses in the M-cell, which implies a change in sensory processing that functionally may increase startle responsiveness.

### Cortisol Potentiates M-Cell Synaptic Responses and Reduces Feedforward Inhibition

Cortisol significantly increased the auditory-evoked response in the goldfish startle circuit, as indicated by the potentiated M-cell PSP (Figure 1A). The amplitude of the PSP is a critical determinant of M-cell activity since it is an integrate—and—fire neuron without an ongoing firing pattern. In addition, the M-cell is relatively inexcitable due to its low input resistance (90–120 kΩ, Neumeister et al., 2008 and this study) and RMP (−80 to −85 mV, Curtin et al., 2013 and this study). As previously shown, even minor changes in PSP amplitude predict changes in startle behavior (Neumeister et al., 2008; Whitaker et al., 2011; Curtin et al., 2013), therefore our results suggest that cortisol increases startle probability. This interpretation is compatible with previous studies in zebrafish showing increased C-start-related activity following stressor exposure (Speedie and Gerlai, 2008). In addition, upregulation of endogenous cortisol release in zebrafish larvae has been shown to increase locomotive activity (De Marco et al., 2013), which is C-start related (Budick and O’Malley, 2000). Finally, an increase in startle responsiveness may also be compatible with exaggerated startle responses in *gr*^s357^ mutant zebrafish larvae, which lack a functional glucocorticoid receptor and over-express cortisol (Griffiths et al., 2012), since the effects of the *gr*^s357^ mutation are limited to the DNA-binding of the glucocorticoid receptor (Ziv et al., 2013). Therefore, the effects of cortisol on cell-signaling discussed later may remain intact and mediate the proposed effects of cortisol on startle.

We considered whether the effects of cortisol on the PSP are produced by increased activity in excitatory auditory afferents and/or by decreasing the effect of inhibitory interneurons. Potentiation of the auditory-evoked M-cell PSP by cortisol was limited to the phase in which chemical excitation and inhibition predominate (Figure 1D), whereas the initial PSP was not significantly affected (Figure 1C). As noted in the results, the initial PSP elicited in response to abrupt sounds is mediated through gap junctions and thus correlates directly with activity in the numerous (75–100) 8th nerve afferences that impinge onto the distal M-cell lateral dendrite (Furukawa and Ishii, 1967; Lin and Faber, 1988a, b; Szabo et al., 2006; Curti and Pereda, 2010). Thus, the lack of an obvious change in the initial PSP suggests that pre-synaptic activity in the auditory afferences was largely unaffected by cortisol. This seems to contradict a study in goldfish in which noise exposure rapidly increases in cortisol levels while reducing auditory brainstem response and increasing threshold, although this study also highlighted the complexity of this possible interaction (Smith et al., 2004). Acute administration of corticosteroids in humans has also been shown to modulate multiple sensory modalities, as measured by auditory hearing threshold (Beckwith et al., 1983; Fehm-Wolfsdorf and Nagel, 1996), taste perception (Henkin, 1970; Fehm-Wolfsdorf et al., 1989), and electroencephalogram (EEG) activity (Born et al., 1988, 1989). The general effect of cortisol on multi-modal sensory pathways supports our interpretation that our results reflect cortisol-induced changes in sensory integration and not differences in activity within the 8th nerve afferences that project to the M-cell, although direct recordings would be required to confirm this possibility.

Eighth nerve afferences also excite a population of interneurons (PHP cells) via monosynaptic projections, which mediate chemical FFI to the M-cell (Faber et al., 1991; Preuss and Faber, 2003; Weiss et al., 2008). The onset and peak of FFI overlaps with the time of cortisol induced cortisol-induced PSP potentiation (Figures 1A, 2B), which suggests that the latter is at least partly mediated by the decrease in FFI following cortisol. In principle, a change in FFI may involve a decrease in either the activation of the pre-synaptic inhibitory neurons (PHP) and/or the efficacy of chemical synapses mediating inhibition at the M-cell dendrite. However, our results favor an effect on the post-synaptic membrane since we did not find evidence for an increase in presynaptic activity in the 8th nerve afferences which activate FFI (see above). As we have previously shown that FFI in the M-cell is strychnine-sensitive and primarily mediated by glycine (Curtin and Preuss, 2015), this interpretation is consistent with results showing that other steroid hormones, including progesterone and to a lesser extent corticosterone reduce glycine inhibition in spinal cord neurons (Wu et al., 1990).

Interestingly, a separate pre-synaptic inhibitory network mediating PPI remained largely unaffected by cortisol, although the observed tonic increase in M-cell membrane excitability (see below) masked the evoked inhibition following a prepulse and produced an apparent overall (ISI non-specific) disruption of PPI. Reduction in behavioral PPI has been observed in humans following cortisol administration and acute stress, which may upregulate cortisol release (Grillon and Davis, 1997; Richter et al., 2011). However, these studies did not test for ISI-specific PPI effects, which is important for tying modulators to PPI deficits (Yeomans et al., 2010; Curtin et al., 2013). Our previous study on the effects of SB-699551, a 5-HT_5a_ antagonist, demonstrated that intrinsic startle circuit excitability modulates PPI in the goldfish (Curtin et al., 2013). Indeed, increased startle associated with increased startle circuit excitability decreases PPI (Blumenthal, 1997; Schicatano et al., 2000). Therefore, we cannot conclusively determine whether these studies contradict or support our findings.

### Cortisol Increases Input Resistance in the Depolarized Post-Synaptic M-Cell Membrane

Cortisol significantly reduced threshold current in the M-cell (Figure 3B), which provides direct evidence that cortisol increases excitability by a post-synaptic mechanism. These effects emerged rapidly (within 10 min) and persisted for an hour before trending towards baseline excitability (Figure 3C). The observed lag time and the emergence of wash out effects provides preliminary evidence of a non-genomic effect of cortisol such as a membrane-bound receptor (Orchinik et al., 1991). However, this conclusion should be interpreted cautiously as our study did not test this concept specifically and 10 min is associated with the upper temporal limit of a non-genomic effect (Makara and Haller, 2001). In addition, administration of glucocorticoid antagonists would be necessary to determine whether our results are specific to the actions of cortisol on a receptor, as demonstrated by Di et al. (2003).

The finding that cortisol increased input resistance only when the M-cell became depolarized (Figure 3E2) indicates that the underlying mechanism may involve the inactivation of a voltage-sensitive membrane conductance. Previous studies demonstrate that cortisol reduces cAMP in tilapia pituitary cells *in vitro* (Borski et al., 1991). In the M-cell, accumulation of cAMP enhances glycine-mediated inhibitory Cl^−^ currents (Wolszon and Faber, 1989) without corresponding changes in RMP, which is consistent with the present study. Our results are also consistent with studies demonstrating a voltage-sensitive action of cortisol reversing noradrenaline-induced excitation in the hippocampus (Joëls and de Kloet, 1989). Indeed, the M-cell membrane contains voltage-sensitive potassium and chloride conductances that increase and decrease input resistance in the depolarized M-cell, respectively (Faber and Korn, 1986, 1987). Thus, the tonic increase in input resistance near threshold might involve either a further inactivation of additional potassium channels and/or the inactivation of normally active chloride channels. Some support for the former comes from *in vitro* studies in the rodent paraventricular nucleus in which cortisol increases excitability by inactivating potassium currents (Zaki and Barrett-Jolley, 2002). However, our result here showed that cortisol had no effect on a voltage-dependent potassium conductance that putatively mediates PPI in the M-cell (Neumeister et al., 2008; Medan and Preuss, 2011, 2014). In contrast, although we do not have direct evidence, the reduction in FFI, which is mediated by glycinergic activation of chloride channels, is consistent with the notion that chloride conductances are affected by cortisol (Curtin and Preuss, 2015). In other words, a cortisol induced inactivation of Cl^−^ channel is a parsimonious explanation for the combined results in our study. Indeed, cortisol induced reduction in chloride conductance has been observed in bullfrog ganglion cells (Ariyoshi and Akasu, 1987), the rodent cortex (Strömberg et al., 2005) and basolateral amygdala (Duvarci and Paré, 2007).

Interestingly, antagonizing serotonin 5a receptor activity increases a chloride dependent tonic inhibition in the M-cell (Curtin et al., 2013). Previous studies have demonstrated the role of serotonin in regulating the stress response (Lanfumey et al., 2008) and administration of the 5-HT_1a_ agonist in salmon upregulates the release of cortisol in a dose-dependent manner (Winberg et al., 1997). Corticosterone has been shown to modulate activity in serotonergic neurons in the dorsal raphe, presumably via activation of G-protein coupled receptors (Wang et al., 2012). These findings raise, albeit hypothetically, the prospect of an interaction of cortisol and serotonin in regulating M-cell excitability.

Physical restraint, which is required for intracellular recording, has been shown to upregulate the release of cortisol in many fish species (Myszkowski et al., 2003; Small, 2003; Bressler and Ron, 2004; Small and Peterson, 2005). In addition, prolonged exposure to MS-222, the general anesthetic used in Experiment 1, also increases plasma concentration of cortisol (Thomas and Robertson, 1991). As such, the experimentally administrated cortisol may add to an already elevated cortisol level in the brain, which potentially masked some of the effects observed between control and drug conditions, i.e., it may underestimate the effects of cortisol observed in our results. In contrast to Experiment 1, MS-222 was substituted with the opioid agonist fentanyl as an analgesic for Experiment 2, an approach used previously to study the effects of cortisol on neural excitability in fish (Remage-Healey and Bass, 2004). Although the responses measured in Experiments 1 and 2 are not directly comparable, we did not find any obvious differences in baseline M-cell membrane properties such as RMP between the experiments (see “Materials and Methods” Section).

The cortisol mediated increase in M-cell excitability highlights the sensitivity of the startle response to acute stress. Indeed, there is a one-to-one relationship between the M-cell AP and the initiation of the startle response (Zottoli, 1977; Weiss et al., 2006) and previous studies showed that changes in M-cell excitability are directly related to changes in startle rate (Preuss and Faber, 2003; Whitaker et al., 2011). Thus, our results provide, albeit indirect, evidence that cortisol likely will increase startle responsiveness in goldfish.

Other processes that mediate relatively complex cognitive and emotional processes, such as learning and memory, also modify the startle response via habituation and sensitization (for review see Schmid et al., 2015). The startle system may therefore receive input from state-dependent processes sensitive to cortisol. Cortisol has been shown to acutely modulate behavior through direct action on neuronal excitability in other species as demonstrated in the inhibition of reproductive behavior in amphibians (Orchinik et al., 1991) and stimulus evoked courtship behavior roughskin newts (Rose et al., 1998), as well as increased vocalization in midshipmen bass (Remage-Healey and Bass, 2004). Broadly, acute stress is thought to promote adaptive, survival-promoting behaviors (McEwen, 1998; Sapolsky, 2002) and given the critical role of the fish start escape response for survival (Korn and Faber, 2005), our results are consistent with the notion that cortisol directly contributes to stress-related changes in behavior. The evolutionarily conserved nature of the auditory startle pathway in mammals and fish (Korn and Faber, 1996), as well as the role of cortisol as the primary corticosteroid in humans, suggests that similar cortisol-sensitive cellular mechanisms may be present in other species including humans.

## Author Contributions

Experiments were performed and data were analyzed by DRB under direct supervision by TP. Manuscript was written by DRB and TP.

## Conflict of Interest Statement

The authors declare that the research was conducted in the absence of any commercial or financial relationships that could be construed as a potential conflict of interest.
